# A comparative systematic review and meta-analysis on the diagnostic accuracy of non-invasive tests for *Helicobacter pylori* detection in elderly patients

**DOI:** 10.3389/fmed.2023.1323113

**Published:** 2023-12-08

**Authors:** Mahmud Omar, Razi Abu-Salah, Reem Agbareia, Yusra Sharif, Roni Levin, Adi Lahat, Kassem Sharif

**Affiliations:** ^1^Faculty of Medicine, Tel Aviv University, Tel Aviv, Israel; ^2^Hebrew University Medical School, Jerusalem, Israel; ^3^Department of Medicine C, Hadassah Medical Centre, Jerusalem, Israel; ^4^Department of Medicine B, Zabludowicz Center for Autoimmune Diseases, Sheba Medical Center, Tel Hashomer, Israel; ^5^Department of Gastroenterology, Sheba Medical Center, Tel Hashomer, Israel

**Keywords:** *H. pylori*, elderly, non-invasive, endoscopy, urea breath test, stool analysis

## Abstract

**Background:**

*Helicobacter pylori* (*H. pylori*) infection, a type I carcinogen, affects approximately 50% of the global population, correlating with various gastric pathologies. Notably, diagnostic sensitivities of non-invasive methods, such as the stool antigen test (HpSA), Serology, and Urea Breath Test (UBT), have been suggested to be less effective in older age groups. This study systematically reviews and meta-analyzes the diagnostic accuracy of these tests within the elderly population.

**Methods:**

A comprehensive literature search was performed across multiple databases, including PubMed, Medline, and Web of Science, up to July 2023. Data were pooled and analyzed using random-effects models. Sensitivity, specificity, and Diagnostic Odds Ratios (DOR) were computed for the tests. Heterogeneity and risk of bias were assessed.

**Results:**

Eight studies involving diverse geographic locations and totaling between 46 and 1,441 participants per study were included. The pooled sensitivity and specificity for HpSA were 72.5 and 94.7%, for Serology 83.7 and 73.3%, and for UBT 96.4 and 88.3%, respectively. DOR for UBT, HpSA, and Serology were 94.5, 47.9, and 14.2, respectively. High levels of heterogeneity were observed across the studies.

**Conclusion:**

UBT and HpSA proved effective for diagnosing *H. pylori* in those over 60, while serology showed lower specificity. Despite methodological variations in available studies, these non-invasive tests offer reliable alternatives, especially for older patients who recently undergone endoscopy or without an indication for it, warranting consideration by healthcare practitioners.

## Introduction

*Helicobacter pylori* (*H. pylori*) infection is ubiquitously prevalent in the human population afflicting close to 50% of individuals globally (1). The epidemiological distribution of *H. pylori* exhibits a marked variability in relation to geographical location, ethnic background, age, and socioeconomic conditions (1). This Gram-negative, type I carcinogen microorganism induces chronic gastric mucosal inflammation, resulting in atrophic and metaplastic alterations. Consequently, *H. pylori* infection is implicated in a myriad of gastric conditions including but not limited to chronic gastritis, peptic ulcers, and gastric-related malignancies (2, 3).

The pathophysiology of the infection is multifaceted, involving intricate interactions between the virulence factors of the bacterium, host immune responses, and various environmental determinants (3). *H. pylori* is diagnosed through a variety of both invasive and non-invasive tests. The choice of diagnostic tool depends on individual patient history and local availability (4). Non-invasive tests, such as the urea breath test (UBT), stool antigen test (HpSA), and serological tests, are generally preferred due to their convenience and non-invasivness (4, 5). However, their diagnostic accuracy can vary, particularly in patients over 65 years, and may also be influenced by conditions such as gastric mucosal atrophy or intestinal metaplasia (4, 6).

The elderly demographic (ages above 65), inherently vulnerable to the detrimental effects of *H. pylori*, encounters a complex clinical scenario (7). The prevalence of *H. pylori* in this age cohort not only exacerbates the predisposition to atrophic gastritis and intestinal metaplasia but also accelerates the trajectory toward gastric malignancies, thereby necessitating a nuanced, age-specific diagnostic and therapeutic approach (8).

Existing guidelines and expert consensuses conspicuously overlook the elderly, often sidelining the unique challenges and considerations pertinent to managing *H. pylori* infections within this population (9). The intricacies of eradication therapies, particularly in the context of potential side effects and the necessity for a meticulous risk–benefit analysis, become paramount, especially given the elderly’s often complex clinical and physiological profiles.

Our meta-analysis seeks to elucidate the accuracy of various diagnostic tests for *H. pylori* within the elderly demographic, aiming to bridge the extant gap in the literature and facilitate the development of robust, individualized management strategies.

## Methods

### Data sources and search strategy

This systematic review and meta-analysis study was prospectively registered at PROSPERO (Registration code: CRD42023463706) and was carried out according to the Preferred Reporting Items for Systematic Reviews and Meta-Analyses (PRISMA) guidelines (10).

A systematic search was conducted on PubMed, Medline, Web of Science, the Cochrane Library, Embase, Google Scholar, and CINAHL from database inception until July 2023 to look for potentially eligible articles. The search strategy was based on the terms appearing in Appendix 1. All retrieval processes were performed manually and independently by two researchers.

### Eligibility criteria

To align with our research objectives, specific eligibility criteria were utilized for study selection. We included observational studies—comprising cohort, case–control, and, where sufficient diagnostic accuracy measures were available, cross-sectional designs—as well as randomized controlled trials. The focus was on studies with a population aged 60 years and above, either by mean age or by exclusive age range of study participants, undergoing evaluation for *H. pylori* infection. The primary outcome was the diagnostic accuracy of non-invasive tests such as the Urea Breath Test, Stool Antigen Test, and serological tests, compared to the gold standard of invasive endoscopy. Studies were excluded if they fell into categories of case reports, case series, letters, comments, editorials, were not published in English, or involved populations younger than 60 years without specific subgroup analysis. Additionally, studies lacking sufficient data to compute sensitivity and specificity were also excluded to maintain analytical rigor.

### Screening and data extraction

A systematic approach was adopted to conduct the study selection process. Two independent reviewers meticulously and manually screened titles and abstracts of retrieved articles against the predefined inclusion criteria, no software was used during the process. Subsequently, full-text articles that showed potential for meeting the eligibility criteria were retrieved for further assessment. During this process, any discrepancies or disagreements between the reviewers were addressed through discussion, and in case of persistent discrepancies, a third reviewer was consulted to ensure objective and unbiased study selection. A standardized data extraction form was employed to facilitate the extraction of relevant data from the studies included. This data extraction form enabled the collection of essential information from each study, ensuring consistency and uniformity in data reporting.

### Quality assessment and methodological evaluation using QUADAS-2

In accordance with rigorous scientific protocols, we employed the Quality Assessment of Diagnostic Accuracy Studies-2 (QUADAS-2) tool to evaluate the methodological integrity of the nine studies incorporated into our meta-analysis (11). Two independent reviewers scrutinized each study across four critical domains: patient selection, index test, reference standard, and flow and timing. The assessments were undertaken with an emphasis on both the risk of bias and applicability, with the first three domains being evaluated for the latter as well. Discrepancies between the reviewers were resolved through consensus-driven discussion.

### Statistical analysis

Statistical analyses were conducted using R Studio. A random-effects model was employed to account for significant heterogeneity observed among the included studies. We computed pooled sensitivity, specificity, and Diagnostic Odds Ratios (DOR) for three diagnostic tests: HpSA, Serology, and UBT. Additionally, we performed a Hierarchical Summary Receiver Operating Characteristic (HSROC) model and generated the corresponding curve to assess the overall diagnostic accuracy. Threshold Effect and Spearman’s Correlation Analysis were also conducted to further evaluate the data. All tests of statistical significance were two-sided, adopting an alpha level of 0.05.

## Results

### Identification of studies

**Figure 1:**
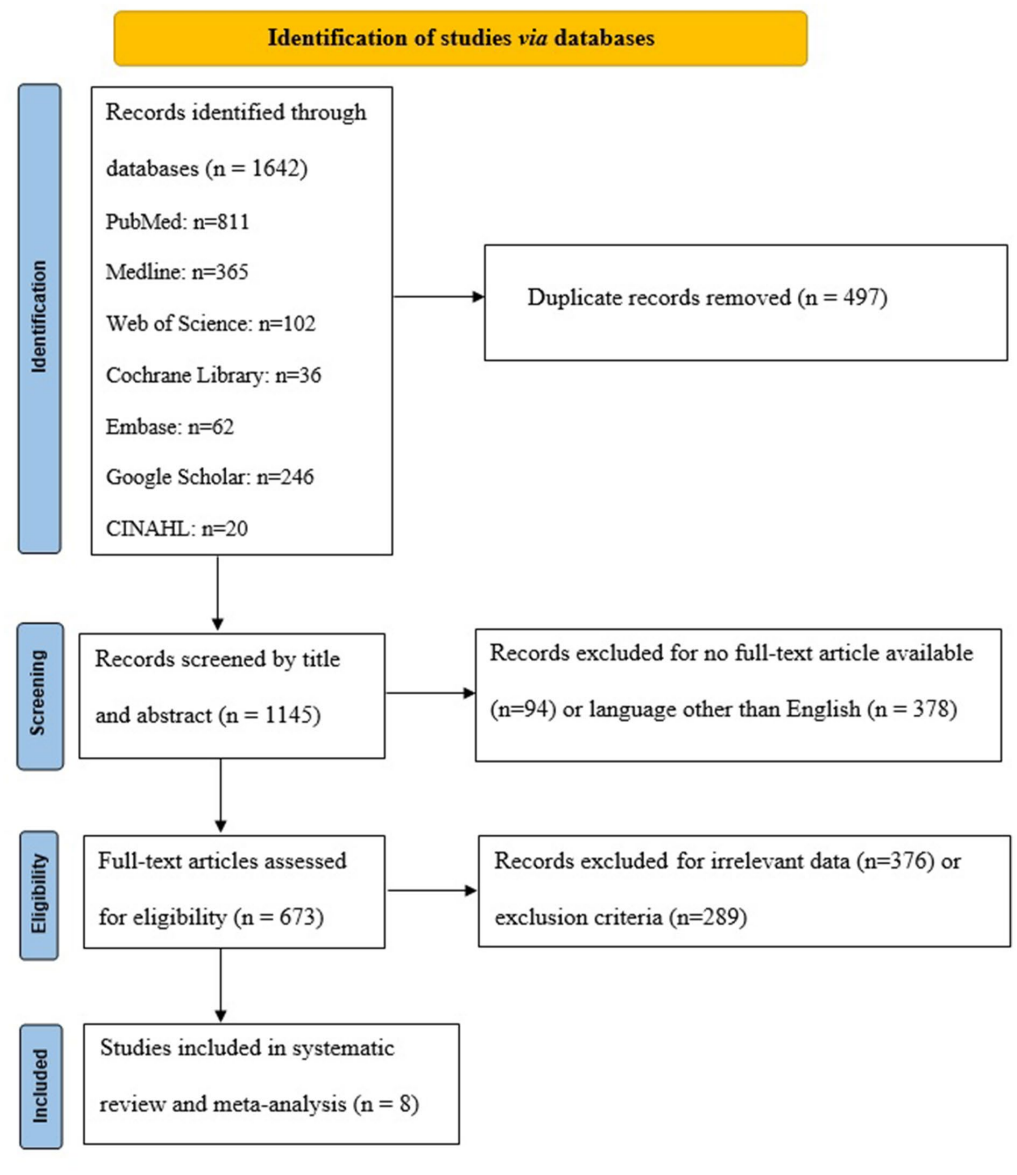
PRISMA flowchart.

A rigorous search strategy was employed across multiple databases to identify studies pertinent to our systematic review and meta-analysis. Initially, the database search yielded 1,642 studies: 811 from PubMed, 365 from Medline, 102 from Web of Science, 36 from Cochrane Library, 62 from Embase, 246 from Google Scholar, and 20 from CINAHL. After eliminating 497 duplicate records, a total of 1,145 studies remained for eligibility assessment. Following an initial screening of titles and abstracts, 673 studies were flagged as potentially eligible and were subjected to full-text review. Upon comprehensive evaluation against our pre-defined eligibility criteria, 8 studies were ultimately included in both the systematic review and meta-analysis. The PRISMA flowchart detailing this process is presented in Figure 1.

### Characteristics of included studies

This meta-analysis incorporates eight studies (12–19), summarized in Table 1. Published between 1991 and 2020, these studies have a broad geographical coverage, including Turkey, Bulgaria, China, Italy, the UK, and Israel. The total number of participants in the individual studies ranged from as few as 46 to as many as 193. The age of the participants varied widely, with study-specific mean ages ranging from 62.6 years to 80.1 years. Both males and females are represented in these studies, although the gender distribution varies among them.

**Table 1:** Summary of the included studies.

Study name and year	Number of participants	Mean age (years)	Type of non-invasive diagnostic test	Reference test	Sensitivity (untreated group)	Specificity (untreated group)
Atli et al. (2012)	100	71 ± 5	[14C] urea breath test	Histology	93.8%	91.4%
Han et al. (2020)	193	77.2 ± 7.8	HpSA	13C UBT	65.1%	98.7%
Inelmen et al. (2004)	122	80.1 ± 6.8	Urea Breath Test, HpSA	Histology	UBT (100%), HpSA (76%)	UBT (85.3%), HpSA (85.3%)
Inelmen et al. (2005)	85	79.2 ± 6.4	HpSA	Histology	76%	93%
Newell et al. (1991)	46	73	[14C] urea breath test	Histology	86.36%	62.5%
Pilotto et al. (2000)	96	77.9	[13C] urea breath test, serology	Histology	[13C] UBT (100%), serology (74.4%)	[13C] UBT (95.74%), serology (59.09%)
Safe et al. (1993)	100	72	Serology (ELISA)	Histology	90%	93%
Shirin et al. (1999)	94	62.6	Serology (FlexPack HP)	Histology	84%	52%

Various non-invasive diagnostic tests for *H. pylori* were employed across the studies, including UBT, HpSA test, and serology. All the studies used endoscopy and histology as reference tests for diagnosing *H. pylori*, except Han et al. (19), which employed [13C] urea breath test as the reference standard. Sensitivity and specificity in the untreated groups varied significantly, with sensitivity ranging from 65.1 to 100%, and specificity ranging from 52 to 98.7%.

The heterogeneity in study design, diagnostic methods, and patient demographics enhances the generalizability and comprehensiveness of our findings. However, it’s worth noting that the follow-up duration was not consistently reported across the studies, with some as short as a three-month period (15).

### Quality assessment and risk of bias of the included studies

**Figure 2:**
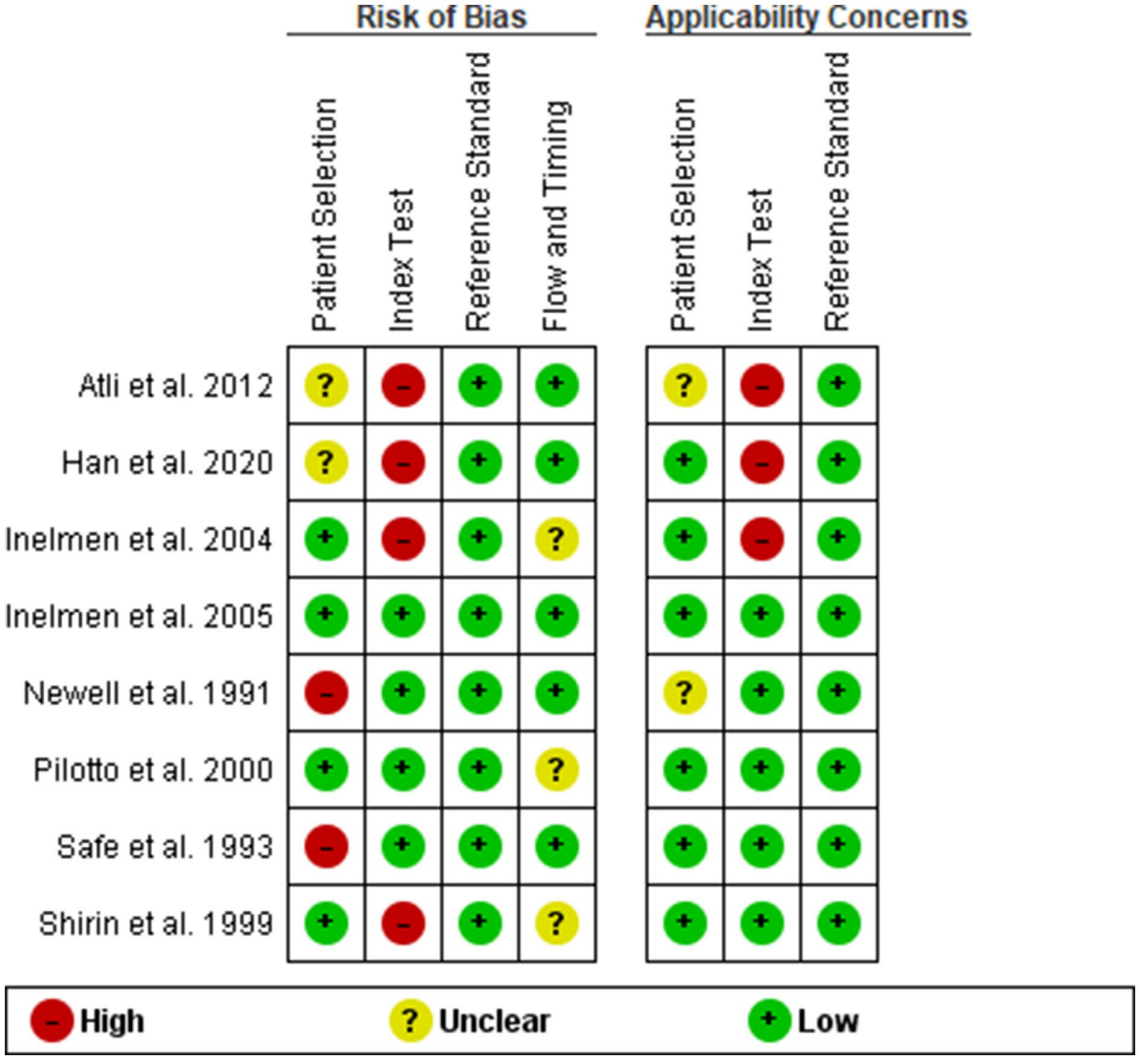
Distribution of risk of bias and applicability concerns across individual domains.

**Figure 3:**
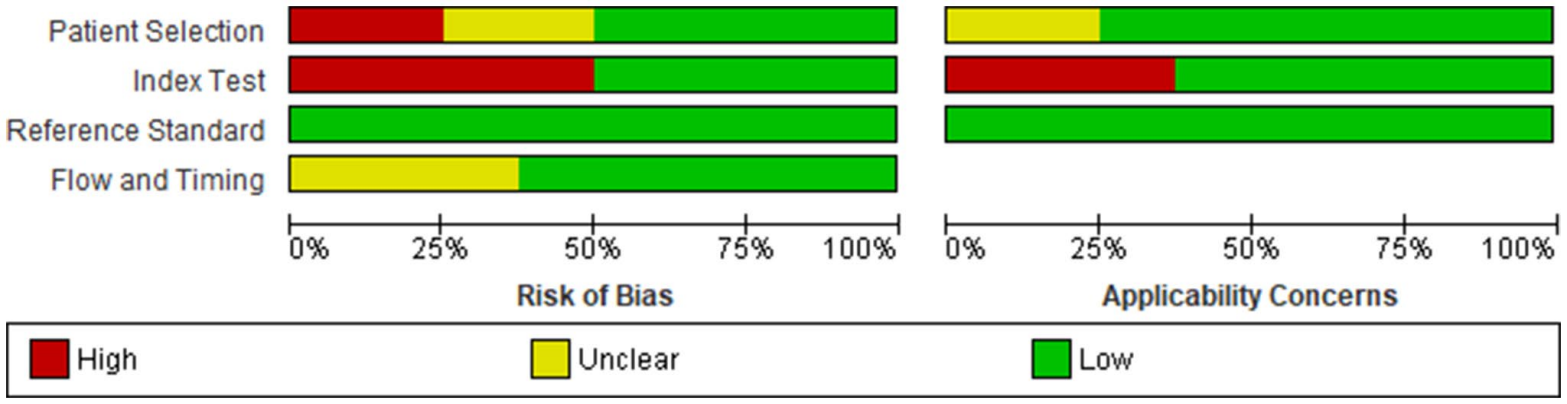
Cumulative assessment of overall risk of bias and applicability concerns across all included studies.

Our analysis revealed that the included studies generally demonstrated a satisfactory level of methodological quality. Nonetheless, specific domains exhibited varying levels of risk. Two studies posed a high risk of bias in the domain of patient selection due to non-consecutive or non-random enrollment (14, 17). A notable concentration of high-risk bias was identified in the index test domain, specifically attributable to pre-defined threshold levels in four out of the eight studies (12, 13, 17, 19). In contrast, no significant risk of bias was detected in the reference standard domain. However, the domain of flow and timing posed an unclear risk in three studies (13, 15, 17), primarily due to the ambiguous time intervals between the administration of the index test and the reference standard. Regarding applicability, the studies were generally robust, although three presented high applicability concerns in the index test domain (12, 13, 19) (Figures 2, 3).

### Random-effects meta-analysis of specificity and sensitivity

#### Diagnostic performance metrics

The diagnostic effectiveness of HpSA, Serology, and UBT in detecting *H. pylori* infection was evaluated through meta-analysis, using the data of the untreated groups. Given the significant heterogeneity among the included studies, random-effects models were employed for the meta-analysis.

### HpSA

**Figure 4:**
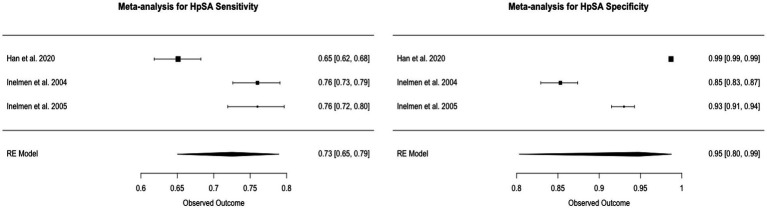
Forest plots of HpSA pooled sensitivity and specificity.

The pooled sensitivity for HpSA was 72.5%, with a 95% Confidence Interval (CI) ranging from 65 to 79%. The pooled specificity was 94.7%, with a 95% CI of 80 to 99%. The I^2 statistics for sensitivity and specificity were 91.73 and 99.54%, respectively, indicating a high level of heterogeneity across the studies. This heterogeneity was statistically significant, with a value of *p* less than 0.0001 for both metrics (Figure 4).

### Serology

**Figure 5:**
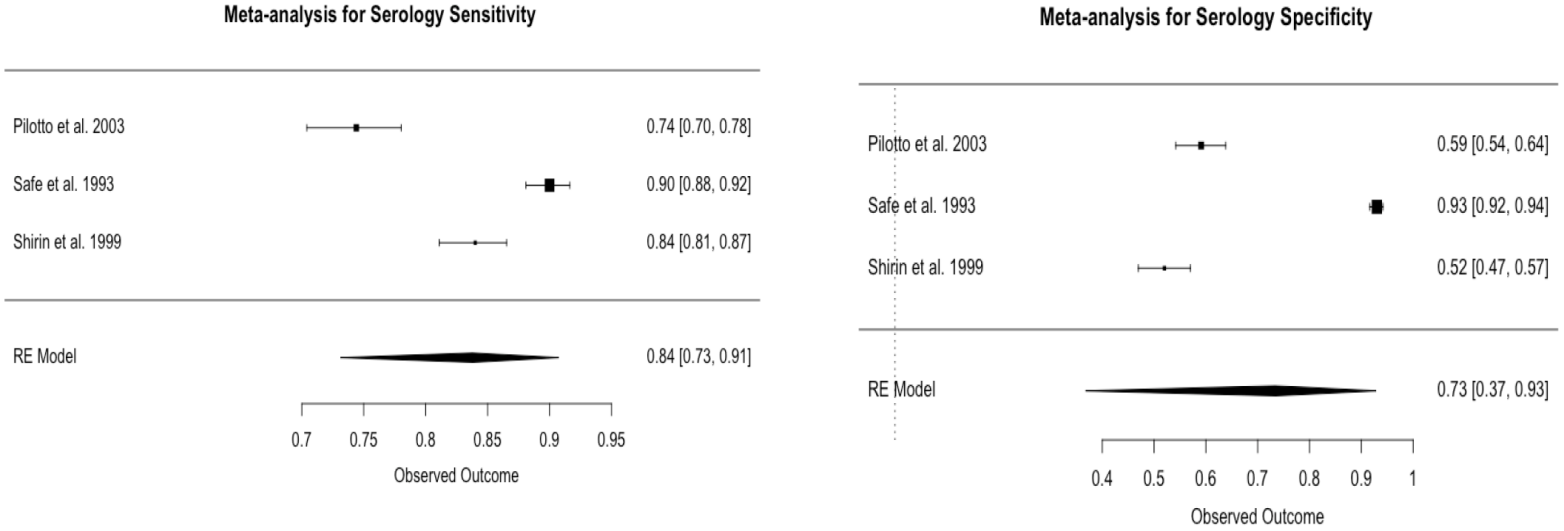
Forest plots of serology pooled sensitivity and specificity.

For Serology tests, the pooled sensitivity was 83.7%, with a 95% CI of 73 to 91%. The pooled specificity was 73.3%, with a 95% CI of 37 to 93%. The I^2 statistics for sensitivity and specificity were 96.77 and 99.45%, respectively, which suggests substantial heterogeneity. This heterogeneity was statistically significant, with a value of *p* less than 0.0001 for both sensitivity and specificity (Figure 5).

### Urea breath test

**Figure 6:**
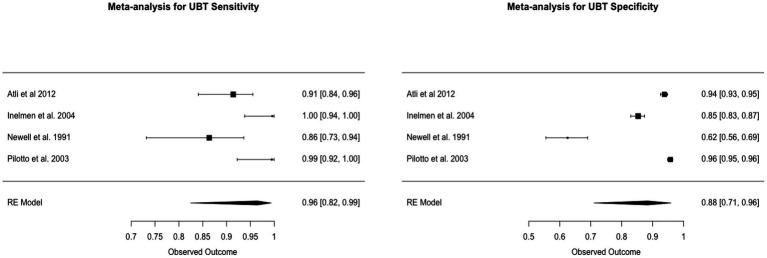
Forest plots of UBT pooled sensitivity and specificity.

The Urea Breath Test (UBT) exhibited a sensitivity of 96.4%, with a 95% CI of 82 to 99%. The specificity was 88.3%, with a 95% CI of 71 to 96%. The I^2 statistics for sensitivity and specificity were 86.14 and 99.15%, respectively, indicating significant heterogeneity. This heterogeneity was confirmed as statistically significant with *p*-values of 0.0149 for sensitivity and less than 0.0001 for specificity (Figure 6).

### Diagnostic odds ratio

We conducted a meta-analysis to estimate the combined Diagnostic Odds Ratios (DOR) for three different tests: HpSA, Serology, and UBT. Diagnostic Odds Ratio (DOR) serves as a single indicator that combines sensitivity and specificity, providing an overall measure of the diagnostic test’s effectiveness. In this study, a higher DOR indicates better discriminative test performance for diagnosing *H. pylori* infection. The results are presented below.

### HpSA test

The meta-analysis yielded a pooled DOR of approximately 47.9 (CI 95%: 14.9–153.2, *p* < 0.001). The studies included in this analysis exhibited significant heterogeneity, with an I^2^ value of 99.25%.

### Serology test

The pooled DOR for Serology tests was approximately 14.2 (CI 95%: 1.7–115.4, *p* = 0.0131). The studies included in this analysis also exhibited significant heterogeneity, with an *I*^2^ value of 99.70%.

### UBT test

For the UBT test, the pooled DOR was approximately 94.5 (CI 95%: 22.4–397.5, *p* < 0.001). The I^2^ statistic was 99.47%, signifying a high level of heterogeneity among the included studies.

**Figure 7:**
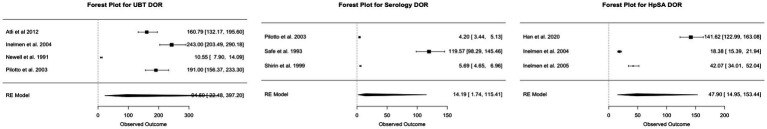
Forest plots of HpSA, serology and UBT pooled diagnostic odds ratio (DOR).

**Figure 8:**
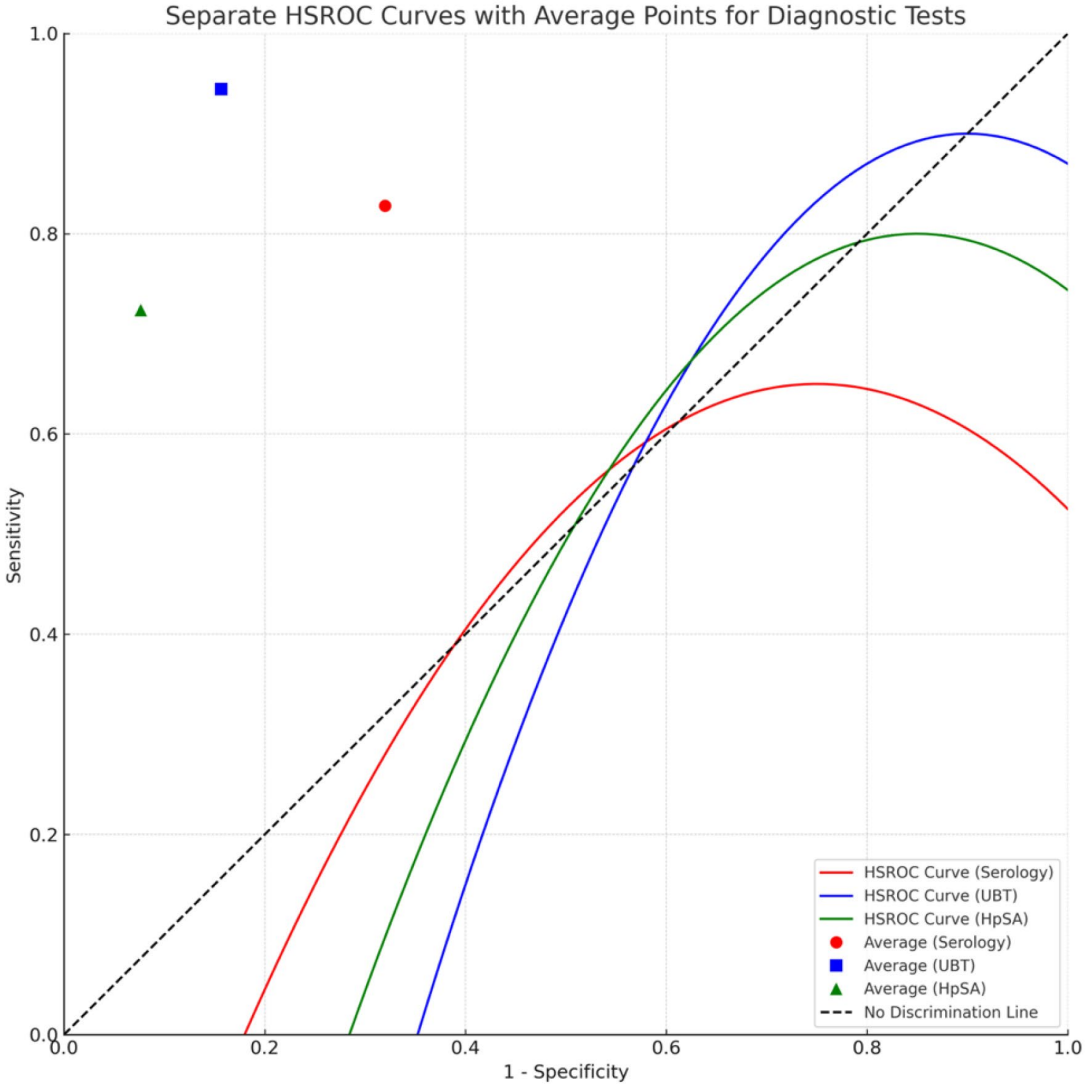
HSROC model illustrating the comparative diagnostic performance of serology, UBT, and HpSA for *Helicobacter pylori* detection. *Points closer to the top-left corner indicate better test performance.

Figure 7 visualizes the DOR’s of the different tests, and Figure 8 represents the findings from the Hierarchical Summary Receiver Operating Characteristic (HSROC) Model Analysis.

### Threshold effect and Spearman’s correlation analysis

In the assessment of the threshold effect using Spearman’s Correlation Analysis, the Serology test exhibited a moderate positive correlation between sensitivity and specificity with a rho value of 0.5, though this was not statistically significant (value of *p* = 1). For the Urea Breath Test (UBT), a rho value of 0.6324555 was observed, indicating a moderate to strong positive correlation between sensitivity and specificity; however, this correlation was not supported by statistical significance (value of *p* = 0.3675). The *Helicobacter pylori* Stool Antigen (HpSA) test demonstrated a strong negative correlation between sensitivity and specificity with a rho value of −0.8660254, yet this too lacked statistical significance (value of *p* = 0.3333).

### Hierarchical summary receiver operating characteristic model analysis

In the meta-analysis, a Hierarchical Summary Receiver Operating Characteristic (HSROC) model was applied to evaluate the diagnostic accuracy of non-invasive tests for *Helicobacter pylori* detection in elderly patients. The model was based on 18 data points derived from the included studies. Using the Restricted Maximum Likelihood (REML) estimation method, the model yielded a log odds ratio estimate of 2.3604, with a 95% confidence interval ranging from 1.7272 to 2.9937. This positive value indicates a consistent trend in the sensitivity and specificity results across the studies. The goodness-of-fit statistics were as follows: Deviance = 7704.8069, AIC = 7708.8069, and BIC = 7710.4733. The estimated between-study variance (sigma^2) was 0.8343, suggesting a moderate heterogeneity among the studies. This was further confirmed by the Cochran’s *Q*-test, which indicated significant variability with a value of *p* of less than 0.0001. The findings from the Hierarchical Summary Receiver Operating Characteristic (HSROC) Model Analysis are visually represented in Figure 8.

## Discussion

### Summary of the main findings

Our meta-analysis assessed the diagnostic accuracy of HpSA, Serology, and Urea Breath Test (UBT) in detecting *H. pylori* infections in the elderly population. The analysis included four studies for UBT, three for HpSA, and three for Serology. The Urea Breath Test showed the highest pooled sensitivity, while HpSA demonstrated superior specificity. In terms of Diagnostic Odds Ratios (DOR), UBT emerged as the most effective diagnostic tool with a DOR of approximately 94.5. HpSA followed with a DOR of around 47.9, and Serology had a DOR of about 14.2. Interestingly, the correlations observed between sensitivity and specificity across these tests suggest a potential deviation from the typical trade-off seen in diagnostic evaluations. The HSROC model further enriched our understanding, indicating a consistent trend in diagnostic accuracy across studies, as evidenced by the positive log odds ratio. However, the significant heterogeneity observed among the studies underscores the importance of individual study contexts and calls for a nuanced interpretation of results. Collectively, our findings spotlight the accuracy of these non-invasive tests in the elderly demographic but also emphasize the variability across studies, underscoring the need for a judicious approach in clinical applications.

### Interpretation of the results and comparison with the general population

The pooled sensitivity and specificity indicate that UBT appears to be the most reliable for diagnosing *H. pylori* among the elderly, particularly given its high DOR, which serves as an overall measure of test effectiveness (20). However, the HpSA test also shows promise, especially in terms of specificity. The DOR values signify the clinical utility of each test, with higher values indicating better diagnostic performance (20). Despite these promising results, the high level of heterogeneity across studies (often exceeding 90%) warrants caution in the generalizability of these findings (21). The *p*-values were consistently less than 0.05 for most metrics, which supports the statistical significance of our results.

Our meta-analysis introduces a nuanced perspective to the burgeoning literature on the diagnostics of *H. pylori* in elderly patients. The pooled results confirm the high sensitivity and specificity of UBT in general, as our study demonstrated that UBT had a pooled sensitivity of 96.4% and specificity of 88.3%, results which align closely with previously published literature declaring UBT as the gold standard with a diagnostic accuracy of around 96% (22). Furthermore, when contrasting our pooled findings with those observed in the general populace, particularly focusing on sensitivity, with our primary objective is to ascertain the accuracy of non-invasive tests in accurately eliminating the occurrence of false negatives. Our results show that the sensitivity of UBT aligns well with the general population’s, as noted by Leal et al.’s meta-analysis, reporting a sensitivity of 95.9% compared to our 96.4%. However, we did observe a slight decrease in specificity, especially in the elderly, with values at 88.3% versus 95.7% (23). It’s worth noting that the specificity can be influenced by age and other physiological factors, and there is evidence suggesting the method might be less specific among elderly patients (24–26). When looking at the pooled results of the HpSA test, the pooled specificity is comparable to results from existing literature on both the general and pediatric populations (27–30). Specifically, our analysis reveals that, although the pooled sensitivity of 72.5% is significantly lower than the 91% sensitivity reported by Gisbert et al. in a meta-analysis encompassing 89 studies, our pooled specificity is comparably analogous, with a value of 94.7% relative to their 93% (4). The high specificity observed might infer that employing this diagnostic tool in older populations holds potential to curtail over-diagnosis and subsequent over-treatment (31). This could further enable its utilization as a confirmatory assay after other diagnostic methodologies characterized by elevated rates of false positives (31, 32). The reduced sensitivity observed in HpSA test can be attributed to multiple factors such as extended gastrointestinal transit time, which could decelerate the passage of bacteria to the colon, and advanced atrophic gastritis, which could diminish test positivity, a condition prevalently observed in the elderly demographic (8, 33–35). As per serology test, we found a pooled sensitivity of 83.7% and specificity of 73.3%. Our results indicate a higher sensitivity but align with the general trend of lower specificity in the elderly (16, 17). In comparison, according to a study that compared the performance of 29 different serological test by Burucoa et al., in the general population, serological tests have shown sensitivities and specificities ranging from 55.6 to 100% and 59.6 to 97.9%, respectively (36). The results may strengthen the idea that *H. pylori* antibody tests may yield high percentage of false positive results in the elderly due to the disappearance of *H. pylori* in advanced gastric mucosal atrophy (37). This implies that in elderly patients with evident signs of *H. pylori* infection, utilizing alternative diagnostics is prudent, as results advocate for serology primarily to exclude, not confirm, infection (38).

### Insights and practical implications

We found that the prevalence of gastric mucosal atrophy, intestinal metaplasia, and other morphological changes in the stomach that increase in older adults, have not impacted the diagnostic accuracy of UBT, when comparing with the general population, as a meta-analysis of UBT diagnostic accuracy shows very similar sensitivity and specificity to our results (6, 8, 35, 39, 40). Our results reveal that such physiological changes may not necessitate the reconsideration of cut-off values in UBT specifically elderly patient, not corroborating previous reports that suggest age-specific adjustments for UBT (24–26, 41, 42), as similar cut-off values were used in the studies assessing UBT diagnostic value (12, 14, 15, 43–45). According to our results, in elderly patients, where there is a high clinical indication of *H. pylori* infection yet a lack of worrisome symptoms or indication for upper GI endoscopy as stated by guidelines (46), the instigation of a non-invasive diagnostic assessment may be a suitable alternative to immediate gastroscopy (47, 48). This approach might be particularly beneficial for elderly patients who present with new and heightened clinical suspicions of *H. pylori* infection and have recently undergone endoscopy, especially if they are within the specified intervals between recommended endoscopies (46, 49), which could offer a pragmatic solution for managing suspected cases efficiently, avoiding unnecessary repeat procedures while ensuring accurate diagnosis and timely intervention. Additionally, even in instances where endoscopy is indicated due to concerning features, identifying, and regularly checking for *H. pylori* via this method can be both time-consuming and expensive (50–52). Thus, employing non-invasive tests can be particularly advantageous in the older population, even when endoscopic examinations are deemed necessary, serving as a cost-effective and efficient alternative for *H. pylori* regular endoscopic screening and detection. We propose that the UBT be administered concomitantly with HpSA testing to minimize the likelihood of over-diagnosis and to exploit the confirmatory value of the elevated specificity exhibited by the HpSA test in this demographic, especially in older patients that have not been lately treated for *H. pylori* (53). The amalgamation of multiple diagnostic tests to precisely identify *H. pylori* infection is a methodology endorsed by Vörhendi et al. within their research conducted on the general population (54).

### Impact of age-related physiological alterations on diagnostic test accuracy

As our research mainly focuses on individuals aged 60 and above, our findings underscore key age-related physiological changes and their potential impact on test sensitivity and specificity. For instance, the decline in gastric emptying, along with hypoxia and increased levels of reactive oxygen species in aging stomachs, could introduce variations in diagnostic accuracy (40, 55–57). This could explain the significant decline in the sensitivity of HpsA test and the specificity of UBT tests among older adults. The decline in the sensitivity of the serological tests could also be attributed to age-related immunosenescence, affecting immunoglobulin titers, and thereby reducing test accuracy in general (19, 37). The reduction of microbial diversity in the stomach, as people age, may also contribute to variations in test accuracy (58). This may also be attributed to the phenomena known as ‘anorexia of aging’ and post-prandial hypotension, which could potentially exacerbate the condition and impede the accuracy of diagnostic procedures. (39, 59).

### Strengths and limitations

One of the major strengths of this meta-analysis is its rigor in methodology, including a comprehensive search strategy and the utilization of dual independent reviewers, thereby minimizing bias, and adhering to PRISMA guidelines (10), with it being the first comprehensive systematic review and meta-analysis in this important topic. However, the significant heterogeneity among studies cannot be overlooked and stands as a substantial limitation (21). The observed extensive heterogeneity could be attributed to numerous elements including the varied prevalence of *H. pylori* across distinct countries and populations and divergent study designs, encompassing discrepancies in the type or protocol of the reference test; for instance, Han et al. utilized 13C Urea Breath Test (UBT) as a reference (2, 8, 19, 21). Furthermore, the exclusion of non-English studies may have constrained the comprehensiveness of our analysis, particularly given the substantial presence of non-English literature identified during our search (10).

## Conclusion and future research

In conclusion, our meta-analysis of 8 studies, which scrutinized three papers on HpSA, three on Serology, and four on UBT, offers a granular and comparative look into the diagnostic accuracy of non-invasive tests for *H. pylori* in individuals aged 60 and over. Our findings imply that the elevated sensitivity of UBT closely mirrors the values found in the general populace. Moreover, our data might infer that merging UBT’s high sensitivity in older individuals, with the HpsA test’s heightened specificity, can produce precise diagnoses, devoid of the peril of false negatives. This might motivate medical practitioners to employ these non-invasive tests in numerous practical scenarios, to identify and address conditions in the elderly effectively.

Future studies should further validate these findings in the elderly through focused Randomized Controlled Trials (RCTs), particularly evaluating the specificity of HpSA as a potential confirmatory test in this age group. Additionally, the impact of commonly prescribed medications—like Proton Pump Inhibitors (PPIs), Non-Steroidal Anti-Inflammatory Drugs (NSAIDs), and antibiotics—on the outcomes of these diagnostic tests needs exploration, enabling a more refined application of diagnostic methods in older populations.

## Data availability statement

The original contributions presented in the study are included in the article/supplementary material, further inquiries can be directed to the corresponding author.

## Author contributions

MO: Investigation, Writing – original draft. RA-S: Methodology, Validation, Writing – review & editing. RA: Conceptualization, Data curation, Validation, Writing – review & editing. YS: Data curation, Validation, Writing – review & editing. RL: Resources, Visualization, Writing – review & editing. AL: Methodology, Software, Validation, Writing – original draft. KS: Writing – original draft.

## Appendix 1

**Table:**

Database	Search strategy
PubMed	#1 *Helicobacter pylori* [MeSH Terms] OR *Helicobacter pylori* [Title/Abstract] OR *H. pylori* [Title/Abstract] #2 Diagnosis [MeSH Terms] OR diagnosis [Title/Abstract] OR detection [Title/Abstract] OR diagnostic [Title/Abstract] #3 older [Title/Abstract] OR elderly [Title/Abstract] #4 #1 AND #2 AND #3
Medline	((((‘*Helicobacter pylori*’) OR ‘*Helicobacter pylori*’ [MeSH Terms]) OR ‘*Helicobacter pylori*’[Title/Abstract])) OR ‘*H. pylori*’ [Title/Abstract]))) AND ((((‘diagnosis’) OR ‘diagnosis’[MeSH Terms]) OR ‘detection’[Title/Abstract]) OR ‘diagnostic’[Title/Abstract])))) AND ((‘older’ [Title/Abstract]) OR ‘elderly’ [Title/Abstract]))
Web of Science	1. *Helicobacter pylori* 2. *H. pylori* 3. Diagnosis 4. Detection 5. Diagnostic 6. Older 7. Elderly 8. 1 or 2 9. 3 or 4 or 5 10. 6 or 7 11. 8 and 9 and 10
Cochrane Library	1. MeSh descriptor: (*Helicobacter pylori*) explode all trees 2. ((*Helicobacter pylori*) or (*H. pylori*): ti, ab, kw 3. Or 1–2 4. MeSh descriptor: (diagnosis) explode all trees 5. ((diagnosis) or (detection) or (diagnosis)): ti, ab, kw 6. Or 4–5 7. ((older) or (elderly)): ti, ab, kw 8. 3 and 6 and 7
Embase	#1 exp *Helicobacter pylori*/ OR exp *H. pylori*/ #2 exp diagnosis/ OR exp. detection/ OR exp. #3 exp older/ or exp. elderly/ #4 #1 and #2 and #3
Google Scholar	“*Helicobacter pylori* (*H. pylori*)” AND (“diagnosis “OR “detection” OR “diagnostic”) AND (“older” OR “elderly”)
CINAHL	#1 *Helicobacter pylori* (mh) OR *H. pylori* #2 diagnosis (mh) OR detection OR diagnostic #3 older OR elderly #4 #1 AND #2 AND #3
